# HIV transmission risk among people living with HIV in Zhejiang Province, China: data from a large cross-sectional study, 2022

**DOI:** 10.3389/fpubh.2025.1550565

**Published:** 2025-04-30

**Authors:** Lin He, Xiaohong Pan, Jiezhe Yang, Jinlei Zheng, Wei Cheng, Chengliang Chai

**Affiliations:** Zhejiang Provincial Center for Disease Control and Prevention, Zhejiang, Hangzhou, China

**Keywords:** human immunodeficiency virus (HIV), people living with HIV, transmission risk, antiretroviral therapy, viral load

## Abstract

**Background:**

Effective management of people living with HIV (PLWH) can block the sexual transmission as there is a zero risk of sexual transmission (by U=U campaign); however, few studies have aimed to addressed the risk of transmission among PLWH in China.

**Method:**

We conducted a cross-sectional survey among PLWH in 2022. PLWH were categorized into four HIV transmission risk groups: on antiretroviral therapy (ART) and HIV viral load (VL) < 50 copies/mL as minimum risk; on ART and 50 ≤ VL < 1,000 copies/mL as low-risk; on ART and VL ≥ 1,000 copies/mL, or on ART but without VL testing as medium-risk; not on ART as high-risk. Multivariable logistic regression was used to identify risk factors associated with risk of HIV transmission.

**Result:**

A total of 39,744 PLWH were enrolled in the study. The proportion of those at risk for HIV transmission was 11.4%: low-risk 3.4%, medium-risk 6.9% and high-risk 1.1%. 33,764 (95.0%) patients were tested for syphilis, of whom 5.6% (1,879) had a current syphilis infection. Multivariable logistic regression analysis showed that compared with patients at minimum risk of transmission, individuals who were male (adjusted odds ratio [aOR]: 1.17, 95% confidence interval [CI]: 1.06–1.29), 16–24 age group (aOR: 2.13, 95% CI: 1.75–2.60), primary school or literate (aOR: 1.50, 95% CI: 1.36–1.65), heterosexual route of HIV infection (aOR: 1.50, 95% CI: 1.38–1.63), with a non-local registered residence (aOR: 1.55, 95% CI: 1.39–1.72), current CD4 + T count ≤200 cells/μL (aOR: 5.03, 95% CI: 4.52–2.59), and follow-up years less than 2 years (aOR: 2.02, 95% CI: 1.77–2.30) were associated with increased odds of HIV transmission. Being married (aOR: 0.62, 95% CI: 0.55–0.69) was associated with a decreased risk of HIV transmission.

**Conclusion:**

We concluded that 8.0% of PLWH were at moderate to high risk for HIV transmission. 88.6% of HIV positive patients in Zhejiang province, China were found to be at minimum risk of HIV transmission. Promoting HIV knowledge and education among younger adults, while linking individuals to ART to reduce their viral load could help reduce the persistently risk of HIV transmission.

## Highlights

- Considerable diagnosed PLWH are at medium to high risk of HIV transmission.
- PLWH being young, less educated, lower CD4 + T count and HIV diagnosis less than 2 years are at higher risk of transmission.
- Targeted interventions reducing HIV transmission should be prioritized for subgroups at higher risk.

## Introduction

The human immunodeficiency virus (HIV) continues to be threat to the public health in China (1). What initially began as an epidemic among injecting drug users has spread to the general population, with sexual transmission now the leading cause of HIV transmission in the country. Of the 107,800 newly diagnosed HIV cases in 2022, more than 97% were infected through sexual transmission (2). Factors such as multiple sexual partners and unprotected sexual behavior among men who have sex with men (MSM) and heterosexual populations have led to the spread of HIV, and are major obstacles to the successful implementation of interventions to prevent and control HIV in high-risk groups (3). It is also widely recognized that these high-risk behaviors can continue at any time after HIV diagnosis (4, 5). Thus, the dynamics of HIV transmission vary according to the characteristics of those infected (6, 7). Various surveys have sought to identify the risk behaviors of infected individuals that may lead to on-going transmission (8). These studies highlight the individual, social, and structural factors that may be helpful in the development of comprehensive interventions in the Chinese context. However, what these studies do not take into account are factors that may be helpful in the identification of potential high-risk individuals.

A number of HIV risk assessment tools have been developed to assist health care providers in the identification of individuals who may be at increased risk for acquiring the disease. These tools could be used not only to discuss risk behaviors, they can also help tailor interventions by stratifying participants into individual risk categories. However these tools are not without limitations. Currently, there is no universally recognized risk assessment tool for HIV transmission, with multiple definitions and strategies for measuring risk of transmission and infectivity. In addition, the evaluation and development of HIV risk assessment methodologies has been limited primarily to high-income countries. In previous studies, the risk of HIV transmission was assessed based on the dose response relationship between HIV patients’ viral load (VL) level and sexual behavior (9); MSM who were living with HIV have been assessed for intentional transmission behavior, VL, anal sex, condom use, depression, and drug abuse (10). HIV phylogenetic analyses have been used to examine HIV transmission patterns among and within risk groups (11). Other studies have defined the risk of HIV transmission as an HIV-infected person who does not use condoms when engaging in sex with an HIV-negative or unknown status sexual partner (12). Specific targets have also been discussed in regards to assessing an individual’s risk of transmitting the disease. A previous study defined HIV transmission risk criteria were defined as follows: HIV VL > 1,500 copies/milliliter (mL) and self-reported risky sexual behavior (13), while another cohort study assessed risk by evaluating types of sexual behavior, condom use, and VL (14). As evidenced by the literature, defining HIV transmission risk remains a challenge, with multiple definitions and tools used around the world.

With the increasing number of people living with HIV (PLWH) and ongoing high-risk behaviors after HIV diagnosis, understanding the characteristics of transmission risks among PLWH can help identify potential individuals contributing to ongoing HIV transmission. In China, however, few studies have focused on developing such risk assessments for the general infected population. Thus, our goal was to define characteristics of high-risk behaviors for HIV in order to identify cases and associated factors that lead to the transmission of the virus. This study can provide a scientific basis for HIV transmission risk assessment methods and help inform future screening efforts.

## Methods

### Sampling and study sites

We conducted a cross-sectional survey among people living with HIV (PLWH) from January to December 2022, all PLWH residing in Zhejiang province in eastern China were identified. Zhejiang province is located in one of the more developed regions of China with 4,279 new HIV infections were diagnosed in 2022. Among these new cases, all were acquired by sexual transmission, 56.7% heterosexual and 41.3% homosexual. The result of implementation of the “95–95-95” strategy in the Zhejiang province were as follows: 85.7% of PLWH were diagnosed, 95.0% of diagnosed patients were on ART, and 94.4% of the patients on ART were viral suppressed (<50 copies/mL).

### Study participants

For this study, PLWH participants were included based on the following criteria: (1) ≥ 15-years-old; (2) followed-up for at least 1 year after HIV diagnosis; (3) residing in Zhejiang province at the end of December 31, 2022.

### Data collection

All diagnosed PLWH registered in the national epidemiological database of Zhejiang province, which tracks all patients diagnosed with HIV in China. Patients received regular follow-up care after HIV diagnosis. Follow-up care was provided at least once per year and consisted primarily of laboratory monitoring, such as CD4 + T-cell testing, viral load testing, behavioral interventions, and treatment information. The local CDC or health care organizations would complete a questionnaire after each follow-up (15, 16), and the survey used data from the one in late December 2022.

### Syphilis testing

Researchers have supported syphilis testing as an objective indicator to assess high-risk behaviors in PLWH (17). Syphilis rapid plasma regain (RPR) positivity may reflect recent high-risk behaviors (18). Therefore, all participants in this study were tested for syphilis between March and December 2022. A venous blood specimen (5 mL) was drawn from each participant following the survey. Syphilis sero-positivity testing was performed using the treponema pallidum particle agglutination assay (TPPA). All syphilis TPPA positive samples were then processed for RPR detection. Those with a positive syphilis RPR status were defined as newly infected individuals (current syphilis).

### Viral load testing

In China, PLWH receive anti-retroviral therapy (ART) with cost. As a part of this national program, patients also receive regular follow-up care, including VL testing once a year after initiating ART. Two sensitive polymerase chain reaction (PCR) assays with a lower limit of detection of 50 copies/mL were used to measure VL. A participant with VL < 50 copies/mL was defined as virally suppressed.

### Categorization of HIV transmission risk

Based on previous literature and discussions with experts, the risk of HIV transmission has been categorized into four groups: minimum risk, low-risk, medium-risk and high-risk (Table 1). In the presence of ART, viral loads can drop to undetectable levels, reducing HIV transmission and infectivity in HIV + patients. Therefore, ART and VL levels were used as the basis for risk stratification.

**Table 1:** The categorization of HIV transmission risk.

Risk type	Definition
Minimum risk	On ART and VL < 50 copies/mL
Low-risk	On ART and 50 copies/mL ≤ VL < 1,000 copies/mL
Medium-risk	On ART and VL ≥ 1,000 copies/mL; or on ART and have not had VL testing
High-risk	Not on ART

HIV, human immunodeficiency virus; ART, antiretroviral therapy; VL, viral load.

### Statistical analysis

Data were double entered using EpiData version 3.1.^1^ After the data were cleaned and verified, statistical analyses were performed. For descriptive analyses, categorical variables were presented as frequencies and proportions, while continuous variables were presented as medians and interquartile ranges (IQRs). Differences in general demographic characteristics of the study population were compared using a Chi-square (χ^2^) test. The mean (standard deviation [SD]) and median (IQR) of the variables were determined. The main outcome was HIV transmission risk, with the “minimum risk” group used as the reference group. Multivariable logistic regression was used to identify risk factors associated with risk of HIV transmission. Factors from the univariate analysis with *p* < 0.10 and/or those previously shown to be associated with differences in HIV transmission risk were included in multivariate regression models. Adjusted odds ratios (AORs) were calculated along with the coinciding 95% confidence intervals (CIs), with the statistical significance level set to *p* ≤ 0.05 and *β* = 0.1. Data were analyzed using SPSS version 19.0 (IBM Corp. Released 2010. IBM SPSS Statistics for Windows, Version 19.0. Armonk, NY: IBM Corp.).

## Results

**Figure 1:**
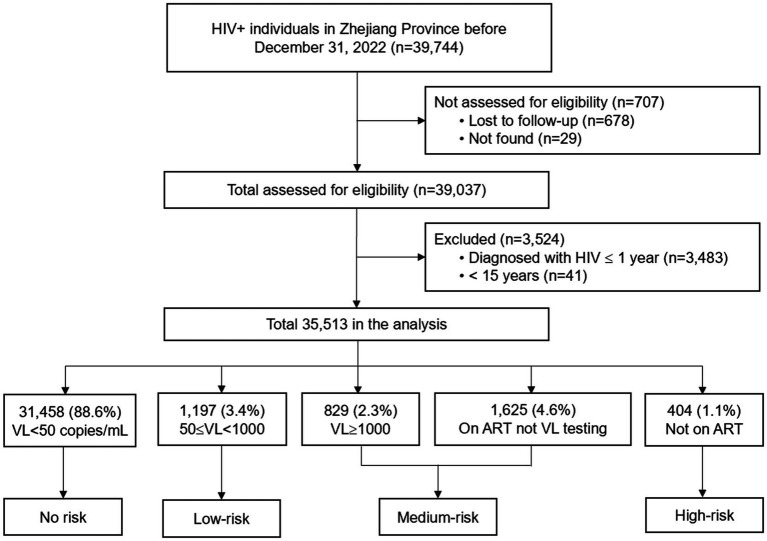
Study design. HIV, human immunodeficiency virus; ART, antiretroviral therapy; VL, viral load.

A total of 39,744 PLHIV residing in Zhejiang Province before December 31, 2022 were recruited. Of these, 35,513 PLWH met the inclusion criteria. Of the participants (and based on ART/VL status), 88.6% were assessed as no-risk individuals, while 11.4% were defined as at risk for HIV transmission: 3.4% low-risk, 6.9% medium-risk, and 1.1% high-risk (Figure 1).

The socio-demographic characteristics of the participants are shown in Table 2. The majority of PLWH were male (82.8%), were aged 25–44 years old (49.5%), married (41.1%), Han ethnicity (96.6%), had at least a junior high school education (76.9%), were heterosexual route of HIV infection (55.0%), originally from local residence (82.7%), had a current CD4 + T count >200 cells/μL (91.8%), and had been followed-up more than 2 years after HIV diagnosis (90.3%). There were significant differences in all variables between the transmission risk groups except sex.

**Table 2:** Socio-demographic characteristics of HIV transmission risk among PLWH residing in Eastern China in 2022.

Variable	N (Column %)	HIV transmission risk				χ^2^	*p*
		Minimum risk N (%)	Low N (%)	Medium N (%)	High N (%)		
Overall	35,513 (100)	31,458 (100)	1,197 (100)	2,454 (100)	404 (100)		
Sex						5.165	0.160
Male	29,422 (82.8)	26,017 (82.7)	1,008 (84.2)	2051 (83.6)	346 (85.6)		
Female	6,091 (17.2)	5,441 (17.3)	189 (15.8)	403 (16.4)	58 (14.4)		
Age (years) IQR 40 (31–51)						119.942	<0.001
16–24	1,268 (3.6)	1,035 (3.3)	52 (4.3)	150 (6.1)	33 (8.2)		
25–34	9,055 (25.5)	7,966 (25.3)	279 (23.3)	686 (28.0)	124 (30.7)		
35–44	8,515 (24.0)	7,632 (24.3)	259 (21.6)	535 (21.8)	89 (22.0)		
45–54	7,541 (21.2)	6,756 (21.5)	250 (20.9)	463 (18.9)	72 (17.8)		
55	9,134 (25.7)	8,069 (25.7)	359 (30.0)	620 (25.3)	86 (21.3)		
Marital status						99.515	<0.001
Single	14,241 (40.1)	12,475 (39.7)	471 (39.3)	1,087 (44.3)	208 (51.5)		
Married	14,603 (41.1)	13,179 (41.9)	487 (40.7)	817 (33.3)	120 (29.7)		
Divorced/separated	6,669 (18.8)	5,804 (18.4)	239 (20.0)	550 (22.4)	76 (18.8)		
Ethnicity						19.156	<0.001
Minority	1,195 (3.4)	1,020 (3.2)	40 (3.3)	120 (4.9)	15 (3.7)		
Han	34,318 (96.6)	30,438 (96.8)	1,157 (96.7)	2,334 (95.1)	389 (96.3)		
Education						87.277	<0.001
Primary school/illiterate	8,202 (23.1)	7,074 (22.5)	331 (27.7)	690 (28.1)	107 (26.5)		
Junior high school	12,129 (34.2)	10,761 (34.2)	410 (34.3)	841 (34.3)	117 (29.0)		
High school and junior college	6,821 (19.2)	6,067 (19.3)	194 (16.2)	472 (19.2)	88 (21.8)		
College	8,361 (23.5)	7,556 (24.0)	262 (21.9)	451 (18.4)	92 (22.8)		
Route of HIV infection						100.240	<0.001
Homosexual	15,531 (43.7)	14,027 (44.6)	480 (40.1)	880 (35.9)	144 (35.6)		
Heterosexual	19,530 (55.0)	17,050 (54.2)	701 (58.6)	1,532 (62.4)	247 (61.1)		
Other	452 (1.3)	381 (1.2)	16 (1.3)	42 (1.7)	13 (3.2)		
Registered residence						130.907	<0.001
Local	29,357 (82.7)	26,120 (83.0)	1,022 (85.4)	1901 (77.5)	314 (77.7)		
Other city of Zhejiang	2,732 (7.7)	2,444 (7.8)	89 (7.4)	175 (7.1)	24 (5.9)		
Other Province	3,424 (9.6)	2,894 (9.2)	86 (7.2)	378 (15.4)	66 (16.3)		
History of STIs before HIV diagnosis						12.973	0.043
No	25,158 (70.8)	22,247 (70.7)	876 (73.2)	1735 (70.7)	300 (74.3)		
Yes	6,569 (18.5)	5,870 (18.7)	199 (16.6)	447 (18.2)	53 (13.1)		
Unknown	3,786 (10.7)	3,341 (10.6)	122 (10.2)	272 (11.1)	51 (12.6)		
Current CD4 + T count (cells/μL) IQR 473 (329–640)						1504.216	<0.001
≤ 200	2,920 (8.2)	2073 (6.6)	165 (13.8)	640 (26.1)	42 (10.4)		
201–499	16,458 (46.3)	14,396 (45.8)	586 (49.0)	1,180 (48.1)	296 (73.3)		
500	16,135 (45.4)	14,989 (47.6)	446 (37.3)	634 (25.8)	66 (16.3)		
Follow-up years after HIV diagnosis IQR 5.8 (3.4–8.6)						387.884	<0.001
< 2	3,812 (10.7)	3,082 (9.8)	254 (21.2)	419 (17.1)	57 (14.1)		
2	7,150 (20.1)	6,194 (19.7)	273 (22.8)	553 (22.5)	130 (32.2)		
4	7,382 (20.8)	6,596 (21.0)	222 (18.5)	491 (20.0)	73 (18.1)		
6	6,441 (18.1)	5,805 (18.5)	171 (14.3)	412 (16.8)	53 (13.1)		
8	4,789 (13.5)	4,365 (13.9)	110 (9.2)	274 (11.2)	40 (9.9)		
10	5,939 (16.7)	5,416 (17.2)	167 (14)	305 (12.4)	51 (12.6)		
Current syphilis infection						173.000	<0.001
Negative	31,855 (89.7)	29,025 (92.3)	1,098 (91.7)	1,620 (66.0)	112 (27.7)		
Positive	1879 (5.3)	1716 (5.5)	52 (4.3)	107 (4.4)	4 (1.0)		
Untesting	1779 (5.0)	717 (2.3)	47 (3.9)	727 (29.6)	288 (71.3)		

HIV, human immunodeficiency virus; IQR, interquartile range; STI, sexually transmitted infection.

### Current syphilis infection among transmission risk groups

As shown in Table 2, 33,764 (95.0%) patients had been tested for syphilis. Among these syphilis testing patients, 5.6% (1,879) were current syphilis infection. The current syphilis infection in different transmission risk group were: 5.5% (1,716/31,458) minimum risk group, 4.3% (52/1,197) low risk group, 4.4% (107/2,454) medium risk and 1.0% (4/404) high risk group. Current syphilis infection had significant differences in transmission risk groups (*p* < 0.001).

### Factors influencing HIV transmission risk

As shown in Table 3, multivariable logistic regression analysis revealed that, compared with patients without transmission risk, individuals who were male (aOR: 1.17, 95% CI: 1.06–1.29), 16–24 age group (aOR: 2.13, 95% CI: 1.75–2.60), primary school or literate (aOR: 1.50, 95% CI: 1.36–1.65), heterosexual route of HIV infection (aOR: 1.50, 95% CI: 1.38–1.63), with a non-local registered residence (aOR: 1.55, 95% CI: 1.39–1.72), current CD4 + T count ≤200 cells/μL (aOR: 5.03, 95% CI: 4.52–2.59), and follow-up years less than 2 year (aOR: 2.02, 95% CI: 1.77–2.30) were associated with increased odds of HIV transmission. Being married (aOR: 0.62, 95% CI: 0.55–0.69) was associated with a decreased risk of HIV transmission in comparison to the single (Table 3).

**Table 3:** Factors associated with HIV transmission risk among PLWH in Eastern China in 2022.

Variables	HIV transmission risk		
	% (n/N)	OR 95% CI	aOR 95% CI
Overall	11.4 (4,055/35513)		
Sex			
Female	10.7 (650/6091)	1.00	1.00
Male	11.6 (3,405/29422)	1.10 (1.00–1.20)	1.17 (1.06–1.29)
Age (years)			
16–24	18.4 (233/1268)	1.71 (1.46–1.99)	2.13 (1.75–2.60)
25–34	12.0 (1,089/9055)	1.04 (0.95–1.13)	1.48 (1.29–1.70)
35–44	10.4 (883/8515)	0.88 (0.80–0.96)	1.27 (1.13–1.43)
45–54	10.4 (785/7541)	0.88 (0.80–0.97)	1.07 (0.96–1.19)
55	11.7 (1,065/9134)	1.00	1.00
Marital status			
Single	12.4 (1766/14241)	1.00	1.00
Married	9.8 (1,424/14603)	0.76 (0.71–0.82)	0.62 (0.55–0.69)
Divorced/separated	13.0 (865/6669)	1.05 (0.97–1.15)	0.88 (0.78–0.99)
Ethnicity			
Minority	14.6 (175/1195)	1.35 (1.14–1.59)	–
Han	11.3 (3,880/34318)	1.00	–
Education			
Primary school/illiterate	13.8 (1,128/8202)	1.50 (1.36–1.65)	1.50 (1.36–1.65)
Junior high school	11.3 (1,368/12129)	1.19 (1.09–1.31)	1.19 (1.09–1.31)
High school and junior college	11.1 (754/6821)	1.17 (1.05–1.30)	1.17 (1.05–1.30)
College	9.6 (805/8361)	1.00	1.00
Route of HIV infection			
Homosexual	9.7 (1,504/15531)	1.00	1.00
Heterosexual	12.7 (2,480/19530)	1.36 (1.27–1.45)	1.50 (1.38–1.63)
Other	15.7 (71/452)	1.74 (1.34–2.25)	1.68 (1.28–2.22)
Registered residence			
Local	11.0 (3,237/29357)	1.00	1.00
Other city of Zhejiang	10.5 (288/2732)	0.95 (0.84–1.08)	0.97 (0.85–1.11)
Other Province	15.5 (530/3424)	1.49 (1.34–1.63)	1.55 (1.39–1.72)
History of STIs before HIV diagnosis			
No	11.6 (2,911/25158)	1.00	–
Yes	10.6 (699/6599)	0.91 (0.83–0.99)	–
Unknown	11.8 (445/3786)	1.02 (0.92–1.13)	–
Current CD4 + T count (cells/μL)			
≤ 200	29.0 (847/2920)	5.34 (4.84–5.91)	5.03 (4.52–5.60)
201–499	12.5 (2062/16458)	1.87 (1.74–2.02)	1.85 (1.71–2.00)
500	7.1 (1,146/16135)	1.00	1.00
Follow-up years after HIV diagnosis			
< 2	19.2 (730/3812)	2.45 (2.17–2.78)	2.02 (1.77–2.30)
2	13.4 (956/7150)	1.60 (1.43–1.79)	1.39 (1.23–1.57)
4	10.6 (786/7382)	1.23 (1.10–1.39)	1.18 (1.04–1.33)
6	9.9 (636/6441)	1.14 (1.01–1.28)	1.14 (1.01–1.28)
8	8.9 (424/4789)	1.01 (0.88–1.15)	1.03 (0.90–1.18)
10	8.8 (523/5939)	1.00	1.00

aOR, adjusted odds ratio; HIV, human immunodeficiency virus; IQR, interquartile range; STI, sexually transmitted infection.

## Discussion

In this study, we analyzed the risk of HIV transmission among a cohort of 35,513 HIV + individuals in eastern China. This study showed that among those diagnosed with HIV more than 1 year ago, the risk of HIV transmission was relatively low, with 88.6% of participants not at risk for HIV transmission. However, among those at risk for HIV transmission, 3.4% were low-risk, 6.9% were medium-risk, and 1.1% were high-risk. In particular, those aged 16–24 years had 2.13 increased odds of being at risk for HIV transmission. Previous research has shown that young people are becoming disproportionately affected by HIV, often underserved, at high risk for loss to follow-up, non-adherence to ART regimens, and are a growing risk group for the continued transmission of the disease (19). A meta-analysis showed a significantly high prevalence of syphilis among PLWH in China from 1999 to 2022, particularly among MSM with HIV (20). Furthermore, a study of China’s HIV care continuum found that only 29% of HIV + people aged 10–19 years and 38% aged 20–29 years achieved viral suppression, compared with 44% of people living with HIV (21). Basic HIV knowledge has been found to be quite low among young people living in China, with more than 85% having no knowledge of HIV prevention (22). Other social, structural, and behavioral barriers have also limited the success of HIV prevention and treatment programs among youth. These factors, combined with low risk perception, may contribute to an increased risk of transmission among this group.

We obtained similar results in other studies (12, 13, 23), those who were less educated, had a CD4 + T cell count of ≤200 cells/μL and follow-up after HIV diagnosis less than 2 years were risk factors associated with HIV transmission risk (Table 3). Those with more education have been found to be less likely to be infected, with more years of education negatively correlated with HIV infection. Inadequate or lack of information prevents individuals from making the appropriate decisions and can lead to potentially risky behaviors (24). Research has shown that increased education is associated with increased testing rates and reduction in HIV-related stigma (24), thereby reducing the risk of transmission. Interesting as well, was that individuals whom were married in our study were at decreased odds of being high-risk for transmission (aOR = 0.62, 95% CI 0.55–0.69). Other studies have reported that being in a marriage or partnership can reduce risky practices and decrease HIV transmission (25, 26). Efforts to promote honest discourse and agency in relationships, while increasing HIV knowledge and education among younger adults in China are needed.

Though ART has been proven to reduce the risk of HIV transmission, it cannot reduce STI infections and has varied results in reducing related sexual risk behaviors. While some previous research has suggested that patients with viral suppression (or those with lower transmission risk) were significantly less likely to engage in high-risk behaviors than those who were not virally suppressed (27, 28), other studies have reported the opposite (13). Some HIV patients on ART were less likely to use condoms, including having unprotected sex with HIV negative persons (13). This is echoed in our study, where 5.6% current syphilis infection, it means those PLWH still exhibited high-risk sexual behaviors. Specifically, of the 1879 patients identified as current syphilis infection, 91.3% were categorized as “no-risk” for HIV transmission, thus suggesting that these virally suppressed patients on ART are exhibiting risky sexual decision-making. This may pose future issues, particularly if these patients experience long-term treatment compliance issues causing their VL to rebound (29, 30). More so, in this study, we found that 5.6% patients current syphilis infection, it means patients still had high-risk transmission behaviors; while in the national epidemiologic of PLWH follow-up system in 2022, only less than 2% patients reported had not used condom when they had sexual behaviors (unpublished surveillance data). This suggests that current syphilis infection status may capture high-risk behaviors of PLWH (20) and is an additional item that should be assessed when considering transmission risk assessments of HIV-positive individuals. This is supported by other studies that found STIs infection was a common risk factor among individuals at high-risk of transmission (12). Therefore, we recommended that PLWH undergo regular syphilis testing as part of their HIV care, coupled with targeted behavioral interventions to promote condom use regardless of viral load status, in order to reduce on-going transmission of the disease.

There were several limitations of this study. First, we defined patients on ART and who did not have VL testing as at medium risk for HIV transmission (Figure 1), which may overestimate the true proportion of medium-risk individuals. Second, this was a cross-sectional study; therefore, the causal factors associated with HIV transmission risk could not be determined. In addition, VL testing was conducted from January to September 2022, while the syphilis testing was conducted in the same year. The data captured may be time-dependent, as changes in VL may lead to changes in the level of transmission risk. Third, it was possible that some PLWH without current syphilis infection engaged in high-risk behaviors but were unwilling to self-report such behaviors during the regular follow up by local CDS or health organizations. The lack of behavioral data such as self-reported condom use, number of sexual partners to corroborate syphilis co-infection as a proxy for high-risk behavior. However, efforts were taken to promote confidentiality and reduce this reporting bias. Additionally, only current syphilis was considered as relevant to high-risk behaviors, rather than including all reportable STIs. It is possible that some patients were infected with other STIs but not infected with current syphilis, which might result in an underestimation of high-risk behavioral transmission risk in this population. Further, depression and other psychosocial factors have been identified as risk factors for HIV transmission (13, 31, 32), but were not included in this study. Further follow-up may be warranted to assess the impact of various STI infections and associated mental health factors on HIV transmission risk. Last, the generalizability of findings to rural or less-developed regions in China, given Zhejiang’s status as a developed province with robust HIV care infrastructure.

## Conclusion

In Zhejiang Province, China 88.6% of HIV positive patients were found to be at no or low-risk for HIV transmission. However, younger participants with male, CD4 + T cell counts less than ≤200 cells/μL, whom were less educated, heterosexual, non-local registered residence and follow-up years after HIV diagnosis less than 2 years were associated with an increased risk of HIV transmission. Promoting HIV knowledge and education among younger adults, while linking individuals to ART to reduce their viral load could help reduce the persistently high risk of HIV transmission in eastern China.

## Data Availability

The raw data supporting the conclusions of this article will be made available by the authors, without undue reservation.

## Footnotes

^1^ http://www.epidata.dk
